# Aqueous solution synthesis of reduced graphene oxide-germanium nanoparticles and their electrical property testing

**DOI:** 10.1186/1556-276X-8-422

**Published:** 2013-10-17

**Authors:** Huabin Yin, Jinmei Luo, Peihui Yang, Pinghe Yin

**Affiliations:** 1 Department of Chemistry, Jinan University, Guangzhou 510632, People's Republic of China; 2 Key Laboratory of Optoelectronic Information and Sensing Technologies of Guangdong Higher Education Institutes, Jinan University, Guangzhou 510632, People's Republic of China

**Keywords:** Aqueous solution synthesis, Reduced graphene oxide-germanium nanoparticles, Dispersibility

## Abstract

Aqueous solution synthesis of reduced graphene oxide-germanium nanoparticles (RGO-GeNPs) was developed using graphene oxide (GO) as stabilizer, which could be conducive to obtain better excellent electrical properties. The information about morphology and chemical composition of the nanomaterials were obtained by TEM, FTIR, EDS, and XRD measurements. Stable aqueous dispersibility of RGO-GeNPs was further improved by poly(sodium 4-styrenesulfonate) (PSS) to obtain amphiphilic polymer-coated RGO-GeNPs (PSS-RGO-GeNPs). A possible mechanism to interpret the formation of RGO-GeNPs was proposed. The as-synthesized RGO-GeNPs showed excellent battery performance when used as an anode material for Li ion batteries. The resulting nanocomposites exhibited high specific capacity and good cycling stability after 80 cycles. This study showed a facile strategy to synthetize graphene and Ge nanocomposites which can be a hopeful anode material with excellent electrical properties for lithium ion batteries.

## Background

With the advent of nanoscience and nanotechnology, semiconductor nanomaterials have received much attention due to their unique physical properties and potential applications in electronics, catalysts, sensors, and optical devices [1]. The group IV semiconductors such as silicon (Si) and germanium (Ge) were unique materials with a wide range of technological applications. Ge or Ge-based nanomaterials have shown valuable physical properties for various applications in solar cells, optoelectronics, bio-imaging, energy conversion, and storage [2].

In recent years, a variety of strategies have been developed to synthesize functional GeNPs physically and chemically [3-7]. Nevertheless, synthesis and application of Ge nanomaterials have suffered from serious limitations such as some stiff experimental conditions, high temperatures, toxic precursors, and complex synthesis process [8]. Furthermore, the application of Ge nanomaterials was often hampered by the aggregation and lowered physical properties, as these facts directly determine the applications of Ge nanomaterials. Though Ge nanomaterials have excited an attractive prospect, the majority of synthetic strategies did not provide facile aqueous solution routes. Moreover, organic and inorganic substances such as PVP [9], (CH_3_)_3_SiCl [10], amino acid [11,12], and graphene [13] have been employed to stabilize Ge nanomaterials and to develop nanomaterials with variant morphologies; these strategies could partly improved the physical performance and stability of the Ge nanomaterials.

Graphene is a single-atom-thick two-dimensional graphitic carbon material, which possesses extraordinary large surface area and chemical stability [14]. Recently, graphene has been used as an excellent substance to acquire variously functional nanomaterials, including graphene-silver nanoparticles [15], graphene-gold nanoparticles [16], graphene-TiO_2_ nanomaterials [17], and graphene-palladium nanoparticles [18]. Recently, some works have reported about synthetizing and studying the electrochemical performance of graphene mixed with Ge nanomaterials [19-23]. For instance, Cheng and Du [22] reported the synthesis of graphene-Ge nanocomposites from expensive GeCl_4_ and graphene oxide as precursor. Although the nanocomposites exhibited a high specific capacity as anode materials for lithium ion batteries (LIBs), this strategy did not acquire a material with long cycle life. Ren et al. [23] reported the synthesis of graphene-Ge nanocomposite by chemical vapor deposition (CVD), which exhibited a good capacity retention behavior and long cycle life as anode materials. However, the strategy did not provide a facile route for synthesis. Moreover, the loss of stability and electrochemical properties often inevitably occurred due to irreversible agglomeration and poor dispersions of graphene-Ge nanocomposites in aqueous solution. Therefore, it was important to find a new synthesized method to prepare water-dispersable Ge nanocomposites with excellent electrical properties.

Herein, we demonstrate a simple and mild method to fabricate the RGO-GeNPs in aqueous solution. Stable aqueous dispersions of nanocomposites were synthesized by the reduction of exfoliated graphite oxide and GeO_2_ precursor. Poly(sodium 4-styrenesulfonate) (PSS) was employed to obtain aqueous dispersibility of PSS-RGO-GeNPs, which was hopeful to further improve its electrochemical properties. The study provided a strategy to synthetize RGO-GeNPs which could be served as promising anode materials for LIBs.

## Methods

### Materials

All reagents in this work were of analytical grade and were used as received without further purification. GeO_2_, PSS (analytically pure), and graphite powders (spectral pure) were purchased from Sinopharm Chemical Reagent Beijing Co. NaBH_4_, the reducing agent, was obtained from Aladdin Chemical Co., Ltd. (China). All the aqueous solutions were prepared with double-distilled water.

### Preparation of RGO-GeNPs and PSS-RGO-GeNPs

Graphene oxide (GO) was prepared by oxidizing natural graphite powder based on a modified Hummers and Offeman method [24] as originally presented by Kovtyukhova et al. [25]. The RGO-GeNPs were synthesized by the following method:10 mL of as-prepared GO supernatant (20 mg/mL) was distributed in 40 mL of ultrapure water to obtain a homogeneous, stable dispersion with the aid of ultrasonication in a water bath (KQ218, 60 W), named 'A solution’. A 0.08 g GeO_2_ was dissolved completely in 10 mL 0.64 M NaOH solution to form Na_2_GeO_3_ liquid precursors, then the pH of the solution was adjusted by 0.5 M HCl solution to be 7 to 8, named 'B solution’. Next, both suspensions were mixed together under constant stirring for 1.0 h. The mixture solution was, in the first, instance put into a water bath at 60°C.Then, under a nitrogen atmosphere and continuous magnetic stirring, fresh NaBH_4_ solution (10 mL, 0.1 M) was added dropwise into the mixture solution. This solution was stirred for 4.0 h more. Afterwards, the solution was dialyzed against deionized water for 3 days. Then, the RGO-GeNPs were freeze-dried and collected in a powder form. When the reduction was carried out in the presence of poly(sodium 4-styrenesulfonate), a stable black PSS-RGO-GeNPs solution was obtained.

### Characterization technique and electrical properties testing

The absorption spectra were recorded on a Cary 5000 UV-visible spectrophotometer (Varian Technology Co., Ltd., Palo Alto, CA, USA). Powder X-ray diffraction (XRD) data were collected using a Bruker D8 Advance X-ray diffractometer (Ettlingen, Germany) equipped with CuKα radiation. The FTIR samples were recorded on Equinox 55 IR spectrometer (Bruker) in the range from 4,000 to 400 cm^-1^ using the KBr-disk method. The TEM micrographs were obtained on Hitachi (H-7650, Tokyo, Japan) for TEM operated at an accelerating voltage at 80 kV. Energy-dispersive X-ray spectroscopy (EDS) was carried out during the transmission electron microscopy (TEM) measurement.

Electrochemical measurements were performed using CR2032 coin-type cells assembled in an argon-filled glove box. For the preparation of RGO-GeNPs, Super carbon black and polyimide (PI) binder (dissolved in *N*-methylpyrrolidone) were mixed in a mass ratio of 85:8:7. The resultant slurry was then uniformly coated on a Cu foil current collector and dried overnight under vacuum. The electrochemical cells were assembled with RGO-GeNP electrode or PSS-RGO-GeNP electrode as cathode, metallic lithium foil as anode, and Celgard 2325 porous film (Charlotte, North Carolina) as separator. The electrolyte used in this work was a solution of 1.2 M LiPF6 dissolved in a mixed solvent of ethylene carbonate (EC) and ethylene methyl carbonate (EMC) (3:7 by volume). In addition, 10 wt% fluoroethylene carbonate (FEC) was added into the above electrolyte as additive. Galvanostatic electrochemical experiments were carried out in a Maccor Series 4000 battery system (Tulsa, OK, USA). The electrochemical tests were performed between 0.01 and 1.5 V vs. lithium at ambient temperature.

## Results and discussion

We have prepared the RGO-GeNPs by a one-step approach. Under the present experimental conditions, GO was suitable for the preparation of RGO-GeNP hybrid because of its large surface area and chemical stability.

### Morphology observation

**Figure 1:**
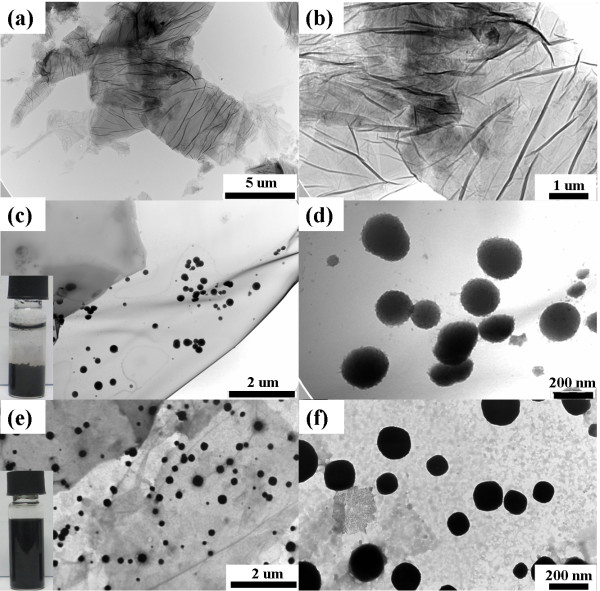
**TEM images of GO, RGO-GeNPs and PSS-RGO-GeNPs at different magnifications. (a**,**b)** GO. **(c**,**d)** RGO-GeNPs. **(e**,**f)** PSS-RGO-GeNPs.

The morphology and microstructures of GO, the RGO-GeNPs, and the PSS-RGO-GeNPs were analyzed by TEM. As shown in Figure 1a,b, the wrinkled structures of GO could not only enlarge its surface area to help absorb and enrich the Ge (IV) ion but also hold the reduced GeNPs and prevent their aggregation. The TEM images of the RGO-GeNPs showed that the GeNPs with diameters of 200 nm were deposited on the basal planes of RGO with less wrinkles in Figure 1c,d. The reasons that caused wrinkle reduction were that the GeNPs adsorbed on the reduced graphene oxide layers resulted in the stretching of its wrinkles, and under the action of reducing agent NaBH4, the hydrophilic group -COOH,-OH, etc. on the surface of GO decreased [26] and the hydrogen bonding lowered, causing a reduction of the wrinkles. When the reduction was carried out in the presence of PSS, the average size of the GeNPs further decreased, and the dispersibility has significantly improved, as shown in Figure 1e,f. An authentic photograph of the PSS-RGO-GeNP solution was given in the inset of Figure 1e. Stable aqueous dispersibility of RGO-GeNPs was further improved in the presence of PSS.

### Formation mechanism of RGO-GeNPs

**Figure 2:**
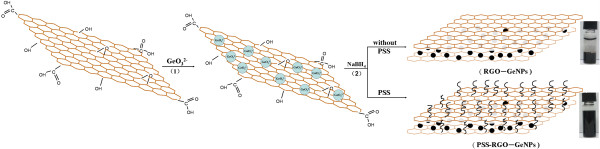
Schematic illustration of the preparation of the RGO-GeNPs and the PSS-RGO-GeNPs.

Figure 2 showed a schematic illustration of the synthesis route for the RGO-GeNPs and PSS-RGO-GeNPs. The formation process of the nanocomposites could be divided into two stages. In the first stage, the oxygen-containing groups on GO could also provide plentiful sites to anchor GeO_3_^2-^ and make them enrich in some places. Consequently, GeO_3_^2-^ was homogeneously dispersed in GO by ultrasonic treatment. In the second stage, the GO nanosheets and the Ge ions could be reduced *in situ* by sodium borohydride, resulting in GeNP loading on graphene nanosheets to fabricate the RGO-GeNPs. Furthermore, stable black aqueous dispersions of PSS-RGO-GeNPs was obtained by coating an amphiphilic PSS.

**Figure 3:**
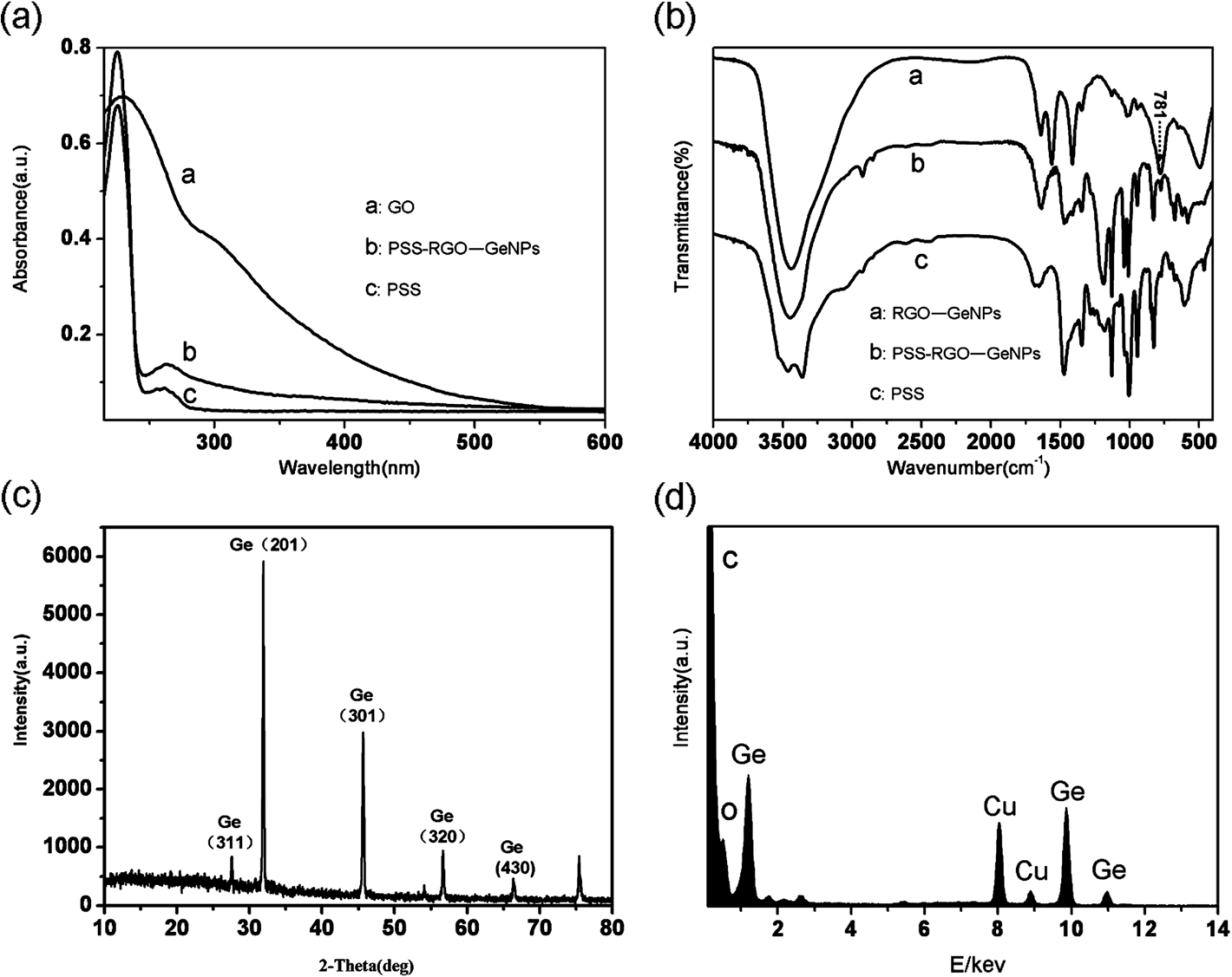
**Chemical composition and structure characterization of the RGO-GeNPs. (a)** Absorption spectrum of the RGO-GeNPs dispersed in aqueous solution. **(b)** FTIR spectra of the RGO-GeNPs and PSS-RGO-GeNPs. **(c)** XRD spectra of the RGO-GeNPs. **(d)** EDS analysis of the RGO-GeNPs.

The stable dispersions of the PSS-RGO-GeNPs were also analyzed by UV–vis spectroscopy. The UV–vis spectrum of the PSS-RGO-GeNPs in water possesses similar features as that of the PSS itself at approximately 262 nm (Figure 3a). A rising absorption edge from 550 nm into the UV gives an evidence of the PSS-RGO-GeNPs. The FTIR spectrum of RGO-GeNPs at 781 cm^-1^ showed the formation of Ge-N bond, clearly indicating the interaction between RGO and GeNPs. Although the FTIR spectrum of PSS-RGO-GeNPs exhibits weak PSS absorption features, it only confirms the presence of the PSS component (Figure 3b). Figure 3c showed a powder XRD pattern for a representative sample of as-synthesized GeNPs, which was in agreement with the standard value for Ge (JCPDS card no. 89–5011). An elemental composition analysis employing EDS showed the presence of a strong signal from the Ge atoms, together with C atom and O from the graphene molecules, whereas a Cu atom signal was ascribed to the supporting grid (Figure 3d).

### Stability test

**Figure 4:**
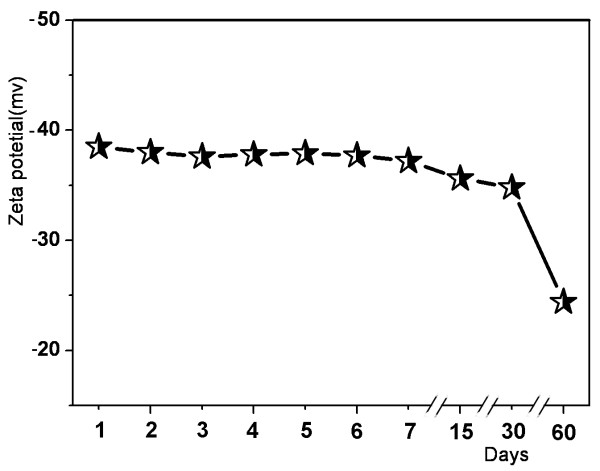
Stability of RGO-GeNPs in aqueous solutions.

Stability is an important issue for the nanomaterials' future application. We measured the zeta potential of the nanocomposites to examine the surface properties and stability of the RGO-GeNPs. Zeta potential is a measurement for electrostatic, charge repulsion or attraction strength between the particles [27]. The American Society of Testing Materials (ASTM) has confirmed that the zeta potential has a close relationship with the degree of dispersion and stability of materials, and the zeta potential can be used as an effective evaluative measure for material stability. Generally, when the zeta potential value of the material is close to ±40 mV, the stability of the material is considered relatively good. As shown in Figure 4, the zeta potential of RGO-GeNPs was -38.7 mV, which just decreased to -36.4 mV after 30 days, explaining a good stability of the RGO-GeNPs. However, the zeta potential of the RGO-GeNPs decreased to -23.3 mV after 60 days, which meant that RGO-GeNPs began to become unstable.

### Electrical properties testing

The theoretical researches showed that Ge exhibits a huge theoretical capacity (1,600 mAhg^-1^) and faster diffusivity of Li compared with Si [22]. Ge can be expected to exhibit excellent electrical properties as anode material for LBIs. Graphene also was a good candidate for Li ion batteries because of its high electrical conductivity, specific wrinkled structures, and flexibility, which made graphene suppress local stress and large volume expansions/shrinkages during a lithiation/delithiation process and alleviate the aggregation or pulverization problems [22]. Therefore, by combining with Ge nanomaterials, the RGO-GeNPs could have enhanced electrical properties, which would be promising materials for various kinds of market-demanded LIBs.

**Figure 5:**
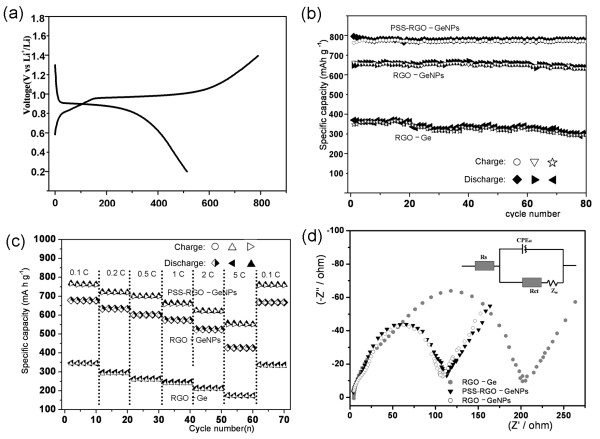
**The electrochemical performance of Ge nanomaterials. (a)** The initial discharge–charge curve of the PSS-RGO-GeNPs cycled between 0 and 1.5 V under a current density of 50 mAg^-1^. **(b)** Cycling behaviors of the PSS-RGO-GeNPs, RGO-GeNPs, and RGO-Ge under a current density of 50 mAg^-1^. Circle, charging of PSS-RGO-GeNPs; filled diamond, discharging of PSS-RGO-GeNPs; empty inverted triangle, charging of RGO-GeNPs; right black triangle, discharging of RGO-GeNPs; empty star, charging of RGO-Ge; left filled triangle, discharging of RGO-Ge. **(c)** Cycling performances of PSS-RGO-GeNPs, RGO-GeNPs, and RGO-Ge under different current densities. Right empty triangle, charging of PSS-RGO-GeNPs; filled triangle, discharging of PSS-RGO-GeNPs; circle, charging of RGO-GeNPs; half-filled diamond, discharging of RGO-GeNPs; left filled triangle, discharging of RGO-Ge. **(d)** Nyquist plots of the electrodes of PSS-RGO-GeNPs, RGO-GeNPs, and RGO-Ge.

The electrochemical performance of the PSS-RGO-GeNPs was tested by galvanostatic discharge/charge technique. Figure 5a showed the discharge/charge voltage profiles cycled under a current density of 50 mAg^-1^ over the voltage range from 0 to 1.5 V vs. Li^+^/Li. The initial discharge and charge specific capacities were 764 and 517 mAhg^-1^, respectively, based on the total mass of the PSS-RGO-GeNPs. The large initial discharge capacity of the nanocomposite could be attributed to the formation of a solid electrolyte interface (SEI) layer.

In our study, the RGO-GeNPs and RGO-Ge were also tested for comparison. As shown in Figure 5b, the PSS-RGO-GeNPs exhibited a higher specific capacity and better cycling stability than RGO-GeNPs and pristine RGO-Ge. The PSS-RGO-GeNPs still retained a reversible capacity of 760 mAhg^-1^ after 80 duty cycles under a current density of 50 mAg^-1^. PSS was employed to obtain aqueous dispersibility of PSS-RGO-GeNPs, which could further improve the electrochemical properties of RGO-GeNPs because of the smaller size and better dispersibility of the GeNPs. The theoretical capacity of PSS-RGO-GeNPs was about two times higher than that of the RGO-Ge. It clearly illustrated that the use of nanosized germanium can effectively overcome the shortcoming of poor cyclability and rapidly declining capacity during the Li uptake and release process.

High rate capabilities and good cycling stability were also observed in the PSS-RGO-GeNPs. As shown in Figure 5c, the PSS-RGO-GeNPs showed a much higher capacity than the RGO-GeNPs and pristine RGO-Ge at different investigated current densities of 0.1 c, 0.2 c, 0.5 c, 1 c, 2 c, and 5 c. Even under the very high current density of 5c, the PSS-RGO-GeNPs still exhibited a favorable specific capacity of 574 mAhg^-1^ after 10 duty cycles. Importantly, the capacity could be recovered to the initial reversible values when the rate was returned to 0.1c, implying their good duty cycling stability and indicating their potential application as promising candidates for the development of high-performance LIBs.

The electrochemical impedance spectra of the PSS-RGO-GeNPs, RGO-GeNPs, and pristine RGO-Ge were demonstrated in Figure 5d. Apparently, the PSS-RGO-GeNP electrode showed a much lower charge transfer resistance *R*_ct_ than the RGO-Ge electrode on the basis of the modified Randles equivalent circuit given in the inset of Figure 5d. This result indicated that the PSS-RGO-GeNP electrode possesses a high electrical conductivity, resulting in the better rate capability and higher reversible capacity in comparison with pristine RGO-Ge.

## Conclusions

In conclusion, we have developed a simple, convenient, and aqueous solution synthesis method to fabricate the RGO-GeNPs under mild conditions. PSS was employed to obtain aqueous dispersibility of PSS-RGO-GeNPs, which was hopeful to further improve its electrical properties. The PSS-RGO-GeNPs exhibited excellent battery performance in comparison with the RGO-GeNPs and pristine RGO-Ge. The resulting nanocomposites exhibit high specific capacity and good cycling stability after 80 cycles, which could be attributed to the electronically conductive and elastic RGO networks, as well as the carbon shells and the small size of the GeNPs. The study provided a strategy to synthetize RGO-GeNPs which could serve as promising anode materials for LIBs.

## Abbreviations

EDS: Energy-dispersive X-ray spectroscopy; FTIR: Fourier transform infrared; GO: Graphene oxide; LIBs: Lithium ion batteries; PSS: Poly(sodium 4-styrenesulfonate); RGO-GeNPs: Reduced graphene oxide-germanium nanoparticles; SEI: Solid electrolyte interface; TEM: Transmission electron microscopy; XRD: X-ray diffraction.

## Competing interests

The authors declare that they have no competing interests.

## Authors’ contributions

PY supervised the study, HY did the experiments, and JL help modify the manuscript. Pinghe Yin provided detection technical support. PY and HY analyzed the data and gave the final approval of the version of the manuscript to be published. All authors read and approved the final manuscript.
